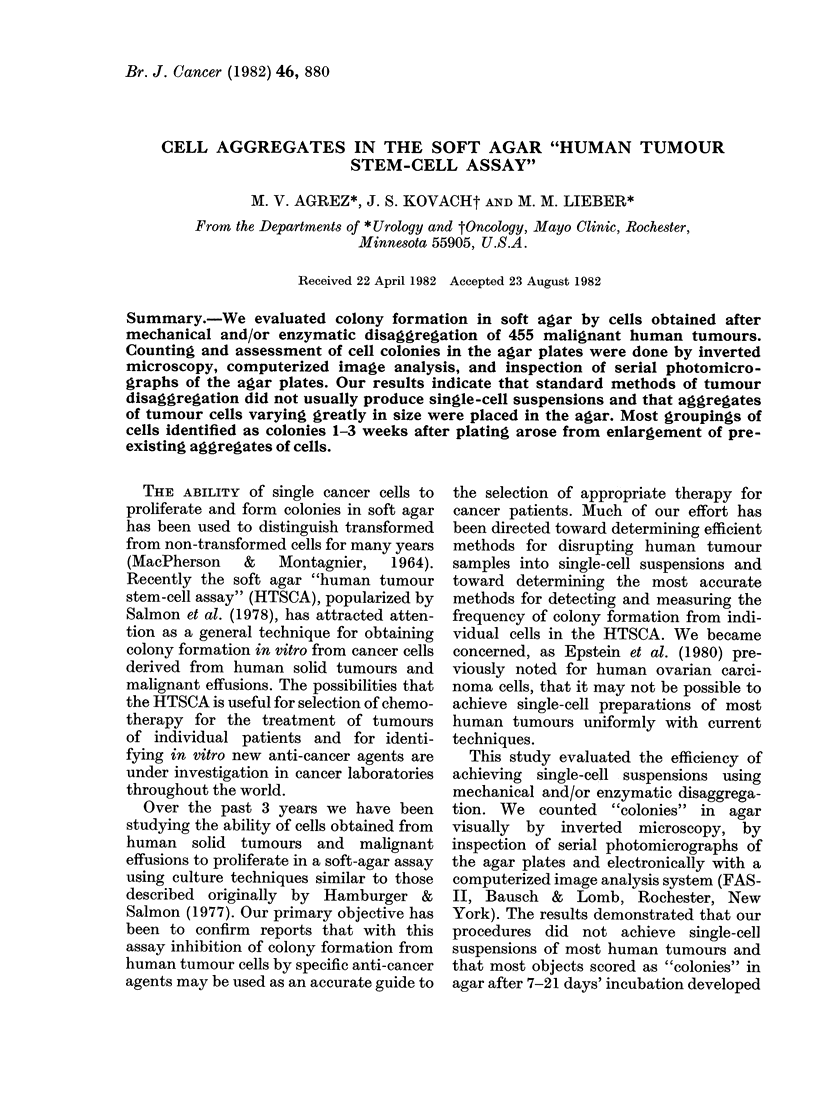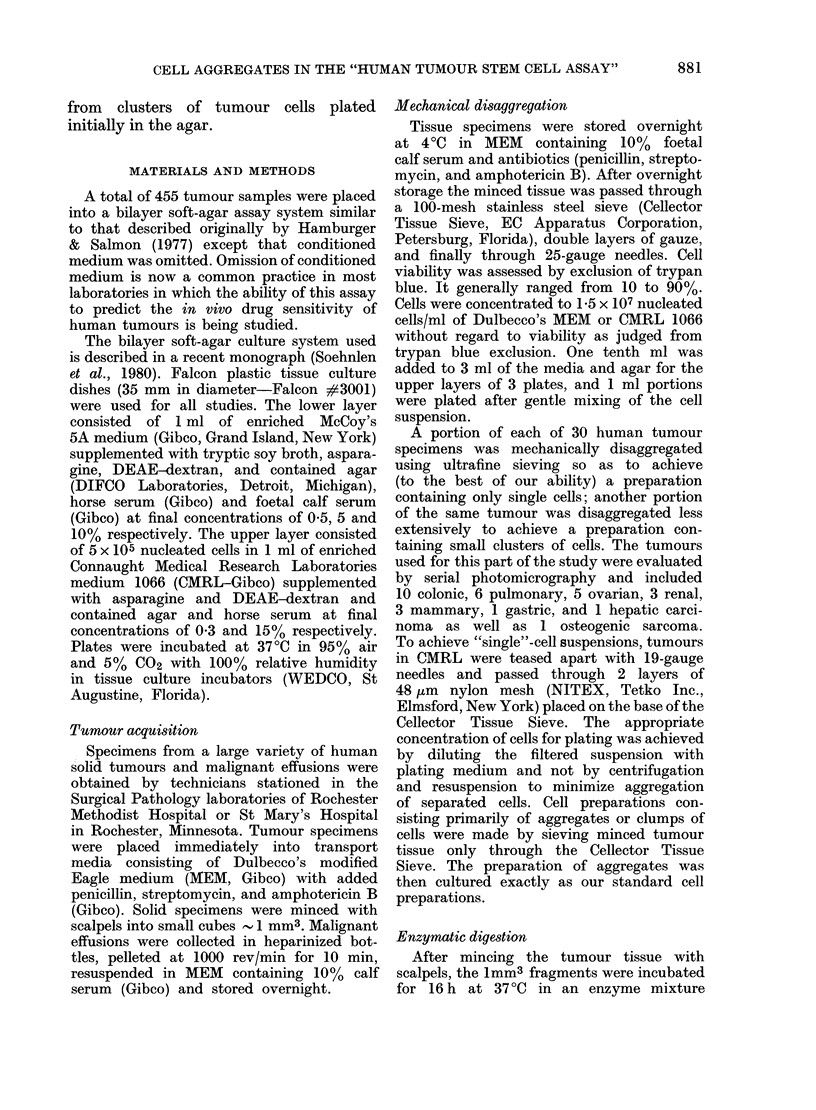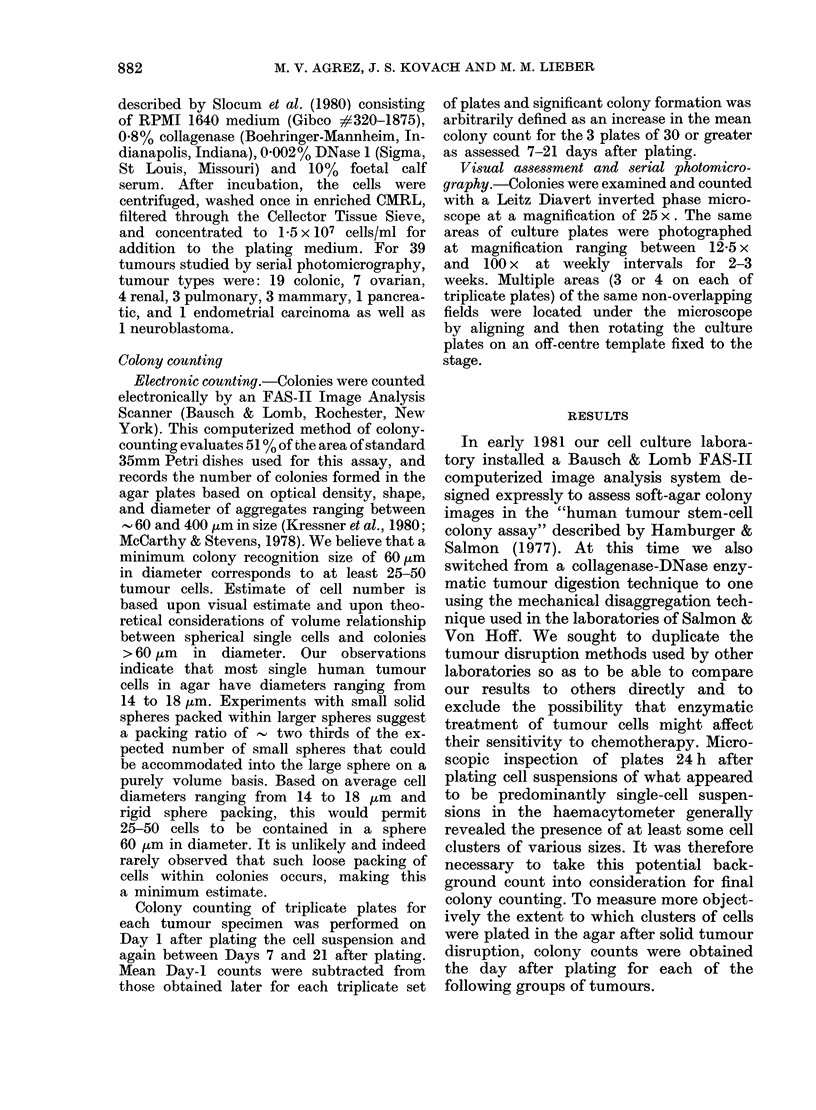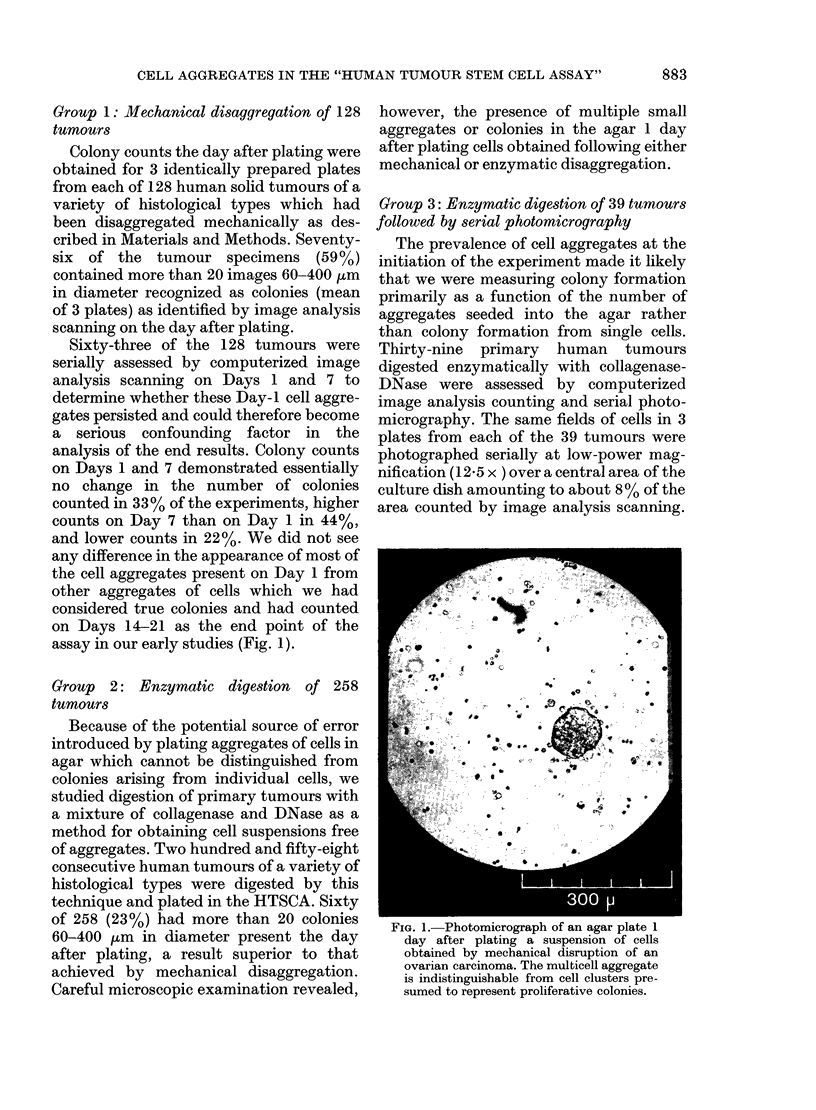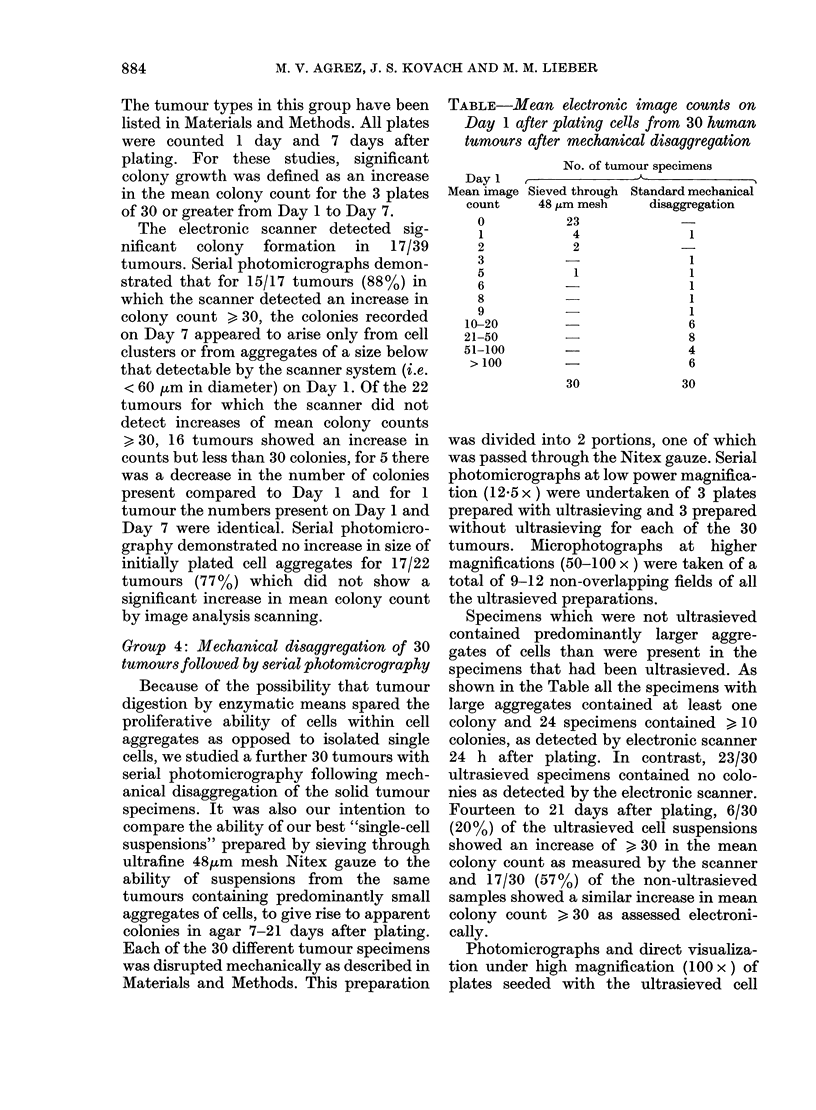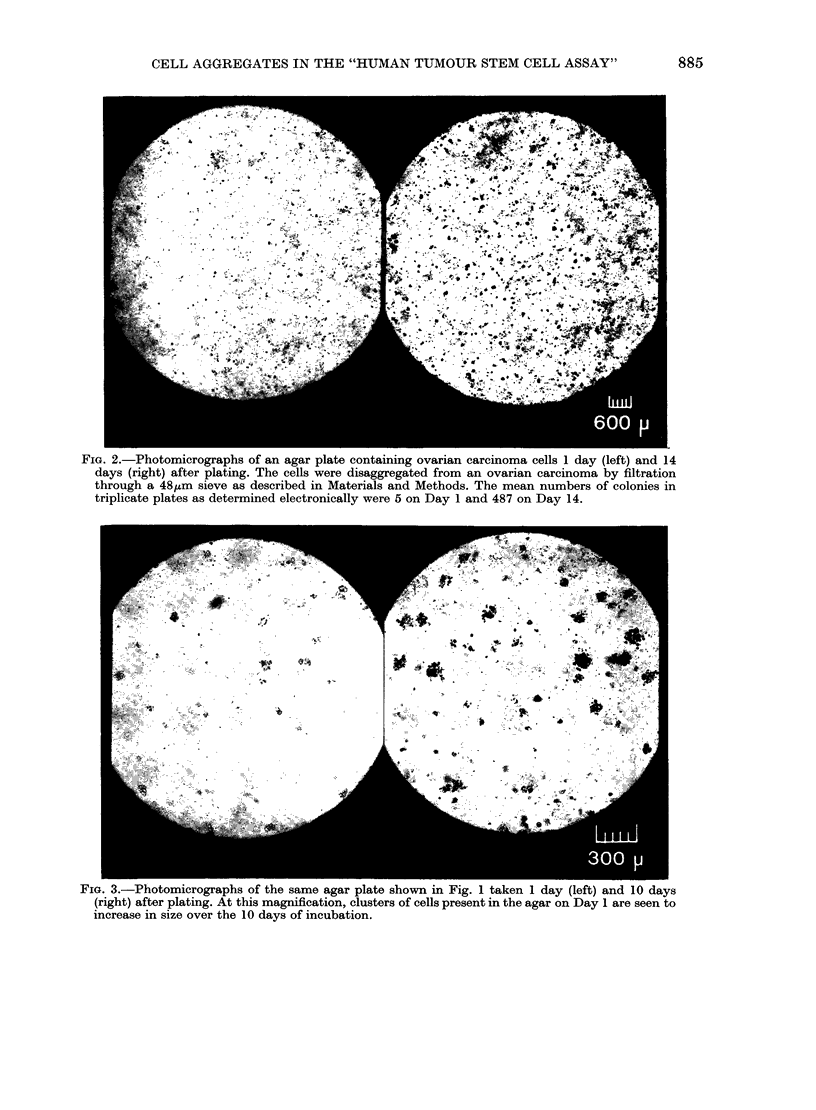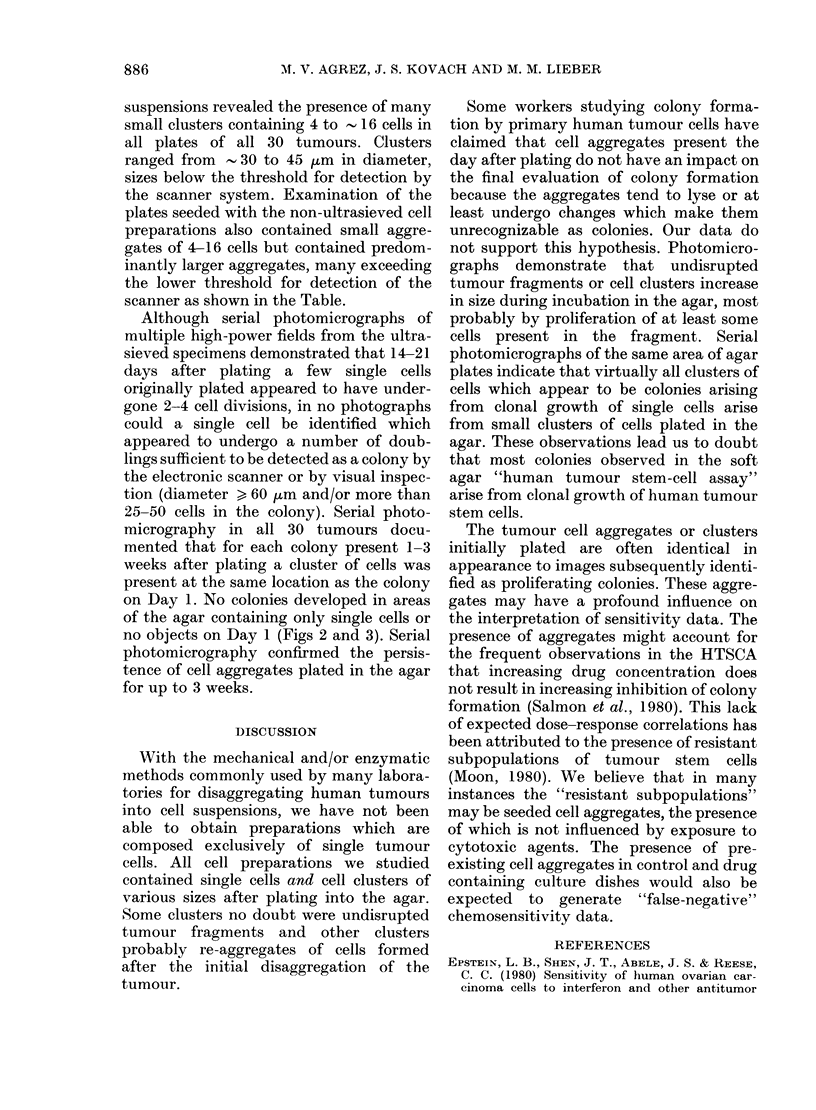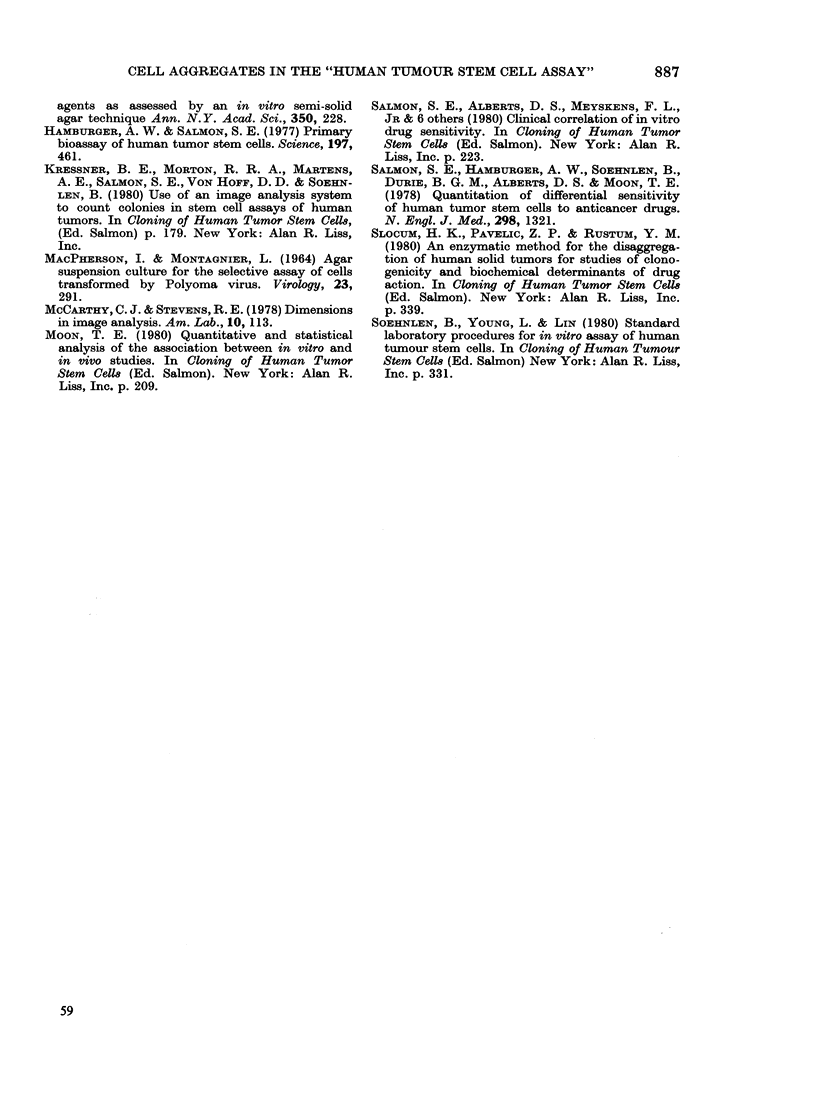# Cell aggregates in the soft agar "human tumour stem-cell assay".

**DOI:** 10.1038/bjc.1982.297

**Published:** 1982-12

**Authors:** M. V. Agrez, J. S. Kovach, M. M. Lieber

## Abstract

**Images:**


*Br. J. Cancer* (1982) **46**, 880

### CELL AGGREGATES IN THE SOFT AGAR "HUMAN TUMOUR STEM-CELL ASSAY"

M. V. AGREZ*, J. S. KOVACH† AND M. M. LIEBER*

*From the Departments of *Urology and †Oncology, Mayo Clinic, Rochester, Minnesota* 55905, *U.S.A.*

Received 22 April 1982 Accepted 23 August 1982

**Summary.—We evaluated colony formation in soft agar by cells obtained after mechanical and/or enzymatic disaggregation of 455 malignant human tumours. Counting and assessment of cell colonies in the agar plates were done by inverted microscopy, computerized image analysis, and inspection of serial photomicrographs of the agar plates. Our results indicate that standard methods of tumour disaggregation did not usually produce single-cell suspensions and that aggregates of tumour cells varying greatly in size were placed in the agar. Most groupings of cells identified as colonies 1–3 weeks after plating arose from enlargement of pre-existing aggregates of cells.**

THE ABILITY of single cancer cells to proliferate and form colonies in soft agar has been used to distinguish transformed from non-transformed cells for many years (MacPherson & Montagnier, 1964). Recently the soft agar "human tumour stem-cell assay" (HTSCA), popularized by Salmon *et al.* (1978), has attracted attention as a general technique for obtaining colony formation *in vitro* from cancer cells derived from human solid tumours and malignant effusions. The possibilities that the HTSCA is useful for selection of chemotherapy for the treatment of tumours of individual patients and for identifying *in vitro* new anti-cancer agents are under investigation in cancer laboratories throughout the world.

Over the past 3 years we have been studying the ability of cells obtained from human solid tumours and malignant effusions to proliferate in a soft-agar assay using culture techniques similar to those described originally by Hamburger & Salmon (1977). Our primary objective has been to confirm reports that with this assay inhibition of colony formation from human tumour cells by specific anti-cancer agents may be used as an accurate guide to the selection of appropriate therapy for cancer patients. Much of our effort has been directed toward determining efficient methods for disrupting human tumour samples into single-cell suspensions and toward determining the most accurate methods for detecting and measuring the frequency of colony formation from individual cells in the HTSCA. We became concerned, as Epstein *et al.* (1980) previously noted for human ovarian carcinoma cells, that it may not be possible to achieve single-cell preparations of most human tumours uniformly with current techniques.

This study evaluated the efficiency of achieving single-cell suspensions using mechanical and/or enzymatic disaggregation. We counted "colonies" in agar visually by inverted microscopy, by inspection of serial photomicrographs of the agar plates and electronically with a computerized image analysis system (FAS-II, Bausch & Lomb, Rochester, New York). The results demonstrated that our procedures did not achieve single-cell suspensions of most human tumours and that most objects scored as "colonies" in agar after 7–21 days' incubation developed from clusters of tumour cells plated initially in the agar.

### MATERIALS AND METHODS

A total of 455 tumour samples were placed into a bilayer soft-agar assay system similar to that described originally by Hamburger & Salmon (1977) except that conditioned medium was omitted. Omission of conditioned medium is now a common practice in most laboratories in which the ability of this assay to predict the *in vivo* drug sensitivity of human tumours is being studied.

The bilayer soft-agar culture system used is described in a recent monograph (Soehnlen *et al.*, 1980). Falcon plastic tissue culture dishes (35 mm in diameter—Falcon #3001) were used for all studies. The lower layer consisted of 1 ml of enriched McCoy's 5A medium (Gibco, Grand Island, New York) supplemented with tryptic soy broth, asparagine, DEAE–dextran, and contained agar (DIFCO Laboratories, Detroit, Michigan), horse serum (Gibco) and foetal calf serum (Gibco) at final concentrations of 0·5, 5 and 10% respectively. The upper layer consisted of $5 \times 10^5$ nucleated cells in 1 ml of enriched Connaught Medical Research Laboratories medium 1066 (CMRL–Gibco) supplemented with asparagine and DEAE–dextran and contained agar and horse serum at final concentrations of 0·3 and 15% respectively. Plates were incubated at 37°C in 95% air and 5% $CO_2$ with 100% relative humidity in tissue culture incubators (WEDCO, St Augustine, Florida).

### *Tumour acquisition*

Specimens from a large variety of human solid tumours and malignant effusions were obtained by technicians stationed in the Surgical Pathology laboratories of Rochester Methodist Hospital or St Mary's Hospital in Rochester, Minnesota. Tumour specimens were placed immediately into transport media consisting of Dulbecco's modified Eagle medium (MEM, Gibco) with added penicillin, streptomycin, and amphotericin B (Gibco). Solid specimens were minced with scalpels into small cubes $\sim 1$ mm³. Malignant effusions were collected in heparinized bottles, pelleted at 1000 rev/min for 10 min, resuspended in MEM containing 10% calf serum (Gibco) and stored overnight.

### *Mechanical disaggregation*

Tissue specimens were stored overnight at 4°C in MEM containing 10% foetal calf serum and antibiotics (penicillin, streptomycin, and amphotericin B). After overnight storage the minced tissue was passed through a 100-mesh stainless steel sieve (Cellector Tissue Sieve, EC Apparatus Corporation, Petersburg, Florida), double layers of gauze, and finally through 25-gauge needles. Cell viability was assessed by exclusion of trypan blue. It generally ranged from 10 to 90%. Cells were concentrated to $1{\cdot}5 \times 10^7$ nucleated cells/ml of Dulbecco's MEM or CMRL 1066 without regard to viability as judged from trypan blue exclusion. One tenth ml was added to 3 ml of the media and agar for the upper layers of 3 plates, and 1 ml portions were plated after gentle mixing of the cell suspension.

A portion of each of 30 human tumour specimens was mechanically disaggregated using ultrafine sieving so as to achieve (to the best of our ability) a preparation containing only single cells; another portion of the same tumour was disaggregated less extensively to achieve a preparation containing small clusters of cells. The tumours used for this part of the study were evaluated by serial photomicrography and included 10 colonic, 6 pulmonary, 5 ovarian, 3 renal, 3 mammary, 1 gastric, and 1 hepatic carcinoma as well as 1 osteogenic sarcoma. To achieve "single"-cell suspensions, tumours in CMRL were teased apart with 19-gauge needles and passed through 2 layers of 48 $\mu$m nylon mesh (NITEX, Tetko Inc., Elmsford, New York) placed on the base of the Cellector Tissue Sieve. The appropriate concentration of cells for plating was achieved by diluting the filtered suspension with plating medium and not by centrifugation and resuspension to minimize aggregation of separated cells. Cell preparations consisting primarily of aggregates or clumps of cells were made by sieving minced tumour tissue only through the Cellector Tissue Sieve. The preparation of aggregates was then cultured exactly as our standard cell preparations.

### *Enzymatic digestion*

After mincing the tumour tissue with scalpels, the 1mm³ fragments were incubated for 16 h at 37°C in an enzyme mixture described by Slocum *et al.* (1980) consisting of RPMI 1640 medium (Gibco #320–1875), 0·8% collagenase (Boehringer-Mannheim, Indianapolis, Indiana), 0·002% DNase 1 (Sigma, St Louis, Missouri) and 10% foetal calf serum. After incubation, the cells were centrifuged, washed once in enriched CMRL, filtered through the Cellector Tissue Sieve, and concentrated to $1{\cdot}5 \times 10^7$ cells/ml for addition to the plating medium. For 39 tumours studied by serial photomicrography, tumour types were: 19 colonic, 7 ovarian, 4 renal, 3 pulmonary, 3 mammary, 1 pancreatic, and 1 endometrial carcinoma as well as 1 neuroblastoma.

*Colony counting*

*Electronic counting.*—Colonies were counted electronically by an FAS-II Image Analysis Scanner (Bausch & Lomb, Rochester, New York). This computerized method of colony-counting evaluates 51% of the area of standard 35mm Petri dishes used for this assay, and records the number of colonies formed in the agar plates based on optical density, shape, and diameter of aggregates ranging between ~60 and 400 μm in size (Kressner *et al.*, 1980; McCarthy & Stevens, 1978). We believe that a minimum colony recognition size of 60 μm in diameter corresponds to at least 25–50 tumour cells. Estimate of cell number is based upon visual estimate and upon theoretical considerations of volume relationship between spherical single cells and colonies >60 μm in diameter. Our observations indicate that most single human tumour cells in agar have diameters ranging from 14 to 18 μm. Experiments with small solid spheres packed within larger spheres suggest a packing ratio of ~ two thirds of the expected number of small spheres that could be accommodated into the large sphere on a purely volume basis. Based on average cell diameters ranging from 14 to 18 μm and rigid sphere packing, this would permit 25–50 cells to be contained in a sphere 60 μm in diameter. It is unlikely and indeed rarely observed that such loose packing of cells within colonies occurs, making this a minimum estimate.

Colony counting of triplicate plates for each tumour specimen was performed on Day 1 after plating the cell suspension and again between Days 7 and 21 after plating. Mean Day-1 counts were subtracted from those obtained later for each triplicate set of plates and significant colony formation was arbitrarily defined as an increase in the mean colony count for the 3 plates of 30 or greater as assessed 7–21 days after plating.

*Visual assessment and serial photomicrography.*—Colonies were examined and counted with a Leitz Diavert inverted phase microscope at a magnification of 25×. The same areas of culture plates were photographed at magnification ranging between 12·5× and 100× at weekly intervals for 2–3 weeks. Multiple areas (3 or 4 on each of triplicate plates) of the same non-overlapping fields were located under the microscope by aligning and then rotating the culture plates on an off-centre template fixed to the stage.

### RESULTS

In early 1981 our cell culture laboratory installed a Bausch & Lomb FAS-II computerized image analysis system designed expressly to assess soft-agar colony images in the "human tumour stem-cell colony assay" described by Hamburger & Salmon (1977). At this time we also switched from a collagenase-DNase enzymatic tumour digestion technique to one using the mechanical disaggregation technique used in the laboratories of Salmon & Von Hoff. We sought to duplicate the tumour disruption methods used by other laboratories so as to be able to compare our results to others directly and to exclude the possibility that enzymatic treatment of tumour cells might affect their sensitivity to chemotherapy. Microscopic inspection of plates 24 h after plating cell suspensions of what appeared to be predominantly single-cell suspensions in the haemacytometer generally revealed the presence of at least some cell clusters of various sizes. It was therefore necessary to take this potential background count into consideration for final colony counting. To measure more objectively the extent to which clusters of cells were plated in the agar after solid tumour disruption, colony counts were obtained the day after plating for each of the following groups of tumours.

*Group* 1: *Mechanical disaggregation of* 128 *tumours*

Colony counts the day after plating were obtained for 3 identically prepared plates from each of 128 human solid tumours of a variety of histological types which had been disaggregated mechanically as described in Materials and Methods. Seventy-six of the tumour specimens (59%) contained more than 20 images 60–400 $\mu$m in diameter recognized as colonies (mean of 3 plates) as identified by image analysis scanning on the day after plating.

Sixty-three of the 128 tumours were serially assessed by computerized image analysis scanning on Days 1 and 7 to determine whether these Day-1 cell aggregates persisted and could therefore become a serious confounding factor in the analysis of the end results. Colony counts on Days 1 and 7 demonstrated essentially no change in the number of colonies counted in 33% of the experiments, higher counts on Day 7 than on Day 1 in 44%, and lower counts in 22%. We did not see any difference in the appearance of most of the cell aggregates present on Day 1 from other aggregates of cells which we had considered true colonies and had counted on Days 14–21 as the end point of the assay in our early studies (Fig. 1).

*Group* 2: *Enzymatic digestion of* 258 *tumours*

Because of the potential source of error introduced by plating aggregates of cells in agar which cannot be distinguished from colonies arising from individual cells, we studied digestion of primary tumours with a mixture of collagenase and DNase as a method for obtaining cell suspensions free of aggregates. Two hundred and fifty-eight consecutive human tumours of a variety of histological types were digested by this technique and plated in the HTSCA. Sixty of 258 (23%) had more than 20 colonies 60–400 $\mu$m in diameter present the day after plating, a result superior to that achieved by mechanical disaggregation. Careful microscopic examination revealed, however, the presence of multiple small aggregates or colonies in the agar 1 day after plating cells obtained following either mechanical or enzymatic disaggregation.

*Group* 3: *Enzymatic digestion of* 39 *tumours followed by serial photomicrography*

The prevalence of cell aggregates at the initiation of the experiment made it likely that we were measuring colony formation primarily as a function of the number of aggregates seeded into the agar rather than colony formation from single cells. Thirty-nine primary human tumours digested enzymatically with collagenase-DNase were assessed by computerized image analysis counting and serial photomicrography. The same fields of cells in 3 plates from each of the 39 tumours were photographed serially at low-power magnification (12·5 × ) over a central area of the culture dish amounting to about 8% of the area counted by image analysis scanning.

FIG. 1.—Photomicrograph of an agar plate 1 day after plating a suspension of cells obtained by mechanical disruption of an ovarian carcinoma. The multicell aggregate is indistinguishable from cell clusters presumed to represent proliferative colonies.

The tumour types in this group have been listed in Materials and Methods. All plates were counted 1 day and 7 days after plating. For these studies, significant colony growth was defined as an increase in the mean colony count for the 3 plates of 30 or greater from Day 1 to Day 7.

The electronic scanner detected significant colony formation in 17/39 tumours. Serial photomicrographs demonstrated that for 15/17 tumours (88%) in which the scanner detected an increase in colony count ≥30, the colonies recorded on Day 7 appeared to arise only from cell clusters or from aggregates of a size below that detectable by the scanner system (*i.e.* <60 μm in diameter) on Day 1. Of the 22 tumours for which the scanner did not detect increases of mean colony counts ≥30, 16 tumours showed an increase in counts but less than 30 colonies, for 5 there was a decrease in the number of colonies present compared to Day 1 and for 1 tumour the numbers present on Day 1 and Day 7 were identical. Serial photomicrography demonstrated no increase in size of initially plated cell aggregates for 17/22 tumours (77%) which did not show a significant increase in mean colony count by image analysis scanning.

*Group 4: Mechanical disaggregation of 30 tumours followed by serial photomicrography*

Because of the possibility that tumour digestion by enzymatic means spared the proliferative ability of cells within cell aggregates as opposed to isolated single cells, we studied a further 30 tumours with serial photomicrography following mechanical disaggregation of the solid tumour specimens. It was also our intention to compare the ability of our best "single-cell suspensions" prepared by sieving through ultrafine 48μm mesh Nitex gauze to the ability of suspensions from the same tumours containing predominantly small aggregates of cells, to give rise to apparent colonies in agar 7–21 days after plating. Each of the 30 different tumour specimens was disrupted mechanically as described in Materials and Methods. This preparation was divided into 2 portions, one of which was passed through the Nitex gauze. Serial photomicrographs at low power magnification (12·5×) were undertaken of 3 plates prepared with ultrasieving and 3 prepared without ultrasieving for each of the 30 tumours. Microphotographs at higher magnifications (50–100×) were taken of a total of 9–12 non-overlapping fields of all the ultrasieved preparations.

TABLE—*Mean electronic image counts on Day 1 after plating cells from 30 human tumours after mechanical disaggregation*

| Day 1 | No. of tumour specimens | |
|---|---|---|
| Mean image count | Sieved through 48 μm mesh | Standard mechanical disaggregation |
| 0 | 23 | — |
| 1 | 4 | 1 |
| 2 | 2 | — |
| 3 | — | 1 |
| 5 | 1 | 1 |
| 6 | — | 1 |
| 8 | — | 1 |
| 9 | — | 1 |
| 10–20 | — | 6 |
| 21–50 | — | 8 |
| 51–100 | — | 4 |
| >100 | — | 6 |
| | 30 | 30 |

Specimens which were not ultrasieved contained predominantly larger aggregates of cells than were present in the specimens that had been ultrasieved. As shown in the Table all the specimens with large aggregates contained at least one colony and 24 specimens contained ≥10 colonies, as detected by electronic scanner 24 h after plating. In contrast, 23/30 ultrasieved specimens contained no colonies as detected by the electronic scanner. Fourteen to 21 days after plating, 6/30 (20%) of the ultrasieved cell suspensions showed an increase of ≥30 in the mean colony count as measured by the scanner and 17/30 (57%) of the non-ultrasieved samples showed a similar increase in mean colony count ≥30 as assessed electronically.

Photomicrographs and direct visualization under high magnification (100×) of plates seeded with the ultrasieved cell

FIG. 2.—Photomicrographs of an agar plate containing ovarian carcinoma cells 1 day (left) and 14 days (right) after plating. The cells were disaggregated from an ovarian carcinoma by filtration through a 48μm sieve as described in Materials and Methods. The mean numbers of colonies in triplicate plates as determined electronically were 5 on Day 1 and 487 on Day 14.

FIG. 3.—Photomicrographs of the same agar plate shown in Fig. 1 taken 1 day (left) and 10 days (right) after plating. At this magnification, clusters of cells present in the agar on Day 1 are seen to increase in size over the 10 days of incubation.

suspensions revealed the presence of many small clusters containing 4 to ~16 cells in all plates of all 30 tumours. Clusters ranged from ~30 to 45 μm in diameter, sizes below the threshold for detection by the scanner system. Examination of the plates seeded with the non-ultrasieved cell preparations also contained small aggregates of 4–16 cells but contained predominantly larger aggregates, many exceeding the lower threshold for detection of the scanner as shown in the Table.

Although serial photomicrographs of multiple high-power fields from the ultrasieved specimens demonstrated that 14–21 days after plating a few single cells originally plated appeared to have undergone 2–4 cell divisions, in no photographs could a single cell be identified which appeared to undergo a number of doublings sufficient to be detected as a colony by the electronic scanner or by visual inspection (diameter $\geqslant 60$ μm and/or more than 25–50 cells in the colony). Serial photomicrography in all 30 tumours documented that for each colony present 1–3 weeks after plating a cluster of cells was present at the same location as the colony on Day 1. No colonies developed in areas of the agar containing only single cells or no objects on Day 1 (Figs 2 and 3). Serial photomicrography confirmed the persistence of cell aggregates plated in the agar for up to 3 weeks.

### DISCUSSION

With the mechanical and/or enzymatic methods commonly used by many laboratories for disaggregating human tumours into cell suspensions, we have not been able to obtain preparations which are composed exclusively of single tumour cells. All cell preparations we studied contained single cells *and* cell clusters of various sizes after plating into the agar. Some clusters no doubt were undisrupted tumour fragments and other clusters probably re-aggregates of cells formed after the initial disaggregation of the tumour.

Some workers studying colony formation by primary human tumour cells have claimed that cell aggregates present the day after plating do not have an impact on the final evaluation of colony formation because the aggregates tend to lyse or at least undergo changes which make them unrecognizable as colonies. Our data do not support this hypothesis. Photomicrographs demonstrate that undisrupted tumour fragments or cell clusters increase in size during incubation in the agar, most probably by proliferation of at least some cells present in the fragment. Serial photomicrographs of the same area of agar plates indicate that virtually all clusters of cells which appear to be colonies arising from clonal growth of single cells arise from small clusters of cells plated in the agar. These observations lead us to doubt that most colonies observed in the soft agar "human tumour stem-cell assay" arise from clonal growth of human tumour stem cells.

The tumour cell aggregates or clusters initially plated are often identical in appearance to images subsequently identified as proliferating colonies. These aggregates may have a profound influence on the interpretation of sensitivity data. The presence of aggregates might account for the frequent observations in the HTSCA that increasing drug concentration does not result in increasing inhibition of colony formation (Salmon *et al.*, 1980). This lack of expected dose–response correlations has been attributed to the presence of resistant subpopulations of tumour stem cells (Moon, 1980). We believe that in many instances the "resistant subpopulations" may be seeded cell aggregates, the presence of which is not influenced by exposure to cytotoxic agents. The presence of preexisting cell aggregates in control and drug containing culture dishes would also be expected to generate "false-negative" chemosensitivity data.

### REFERENCES

Epstein, L. B., Shen, J. T., Abele, J. S. & Reese, C. C. (1980) Sensitivity of human ovarian carcinoma cells to interferon and other antitumor agents as assessed by an *in vitro* semi-solid agar technique *Ann. N.Y. Acad. Sci.*, **350**, 228.

HAMBURGER, A. W. & SALMON, S. E. (1977) Primary bioassay of human tumor stem cells. *Science*, **197**, 461.

KRESSNER, B. E., MORTON, R. R. A., MARTENS, A. E., SALMON, S. E., VON HOFF, D. D. & SOEHNLEN, B. (1980) Use of an image analysis system to count colonies in stem cell assays of human tumors. In *Cloning of Human Tumor Stem Cells*, (Ed. Salmon) p. 179. New York: Alan R. Liss, Inc.

MACPHERSON, I. & MONTAGNIER, L. (1964) Agar suspension culture for the selective assay of cells transformed by Polyoma virus. *Virology*, **23**, 291.

MCCARTHY, C. J. & STEVENS, R. E. (1978) Dimensions in image analysis. *Am. Lab.*, **10**, 113.

MOON, T. E. (1980) Quantitative and statistical analysis of the association between *in vitro* and *in vivo* studies. In *Cloning of Human Tumor Stem Cells* (Ed. Salmon). New York: Alan R. Liss, Inc. p. 209.

SALMON, S. E., ALBERTS, D. S., MEYSKENS, F. L., JR & 6 others (1980) Clinical correlation of in vitro drug sensitivity. In *Cloning of Human Tumor Stem Cells* (Ed. Salmon). New York: Alan R. Liss, Inc. p. 223.

SALMON, S. E., HAMBURGER, A. W., SOEHNLEN, B., DURIE, B. G. M., ALBERTS, D. S. & MOON, T. E. (1978) Quantitation of differential sensitivity of human tumor stem cells to anticancer drugs. *N. Engl. J. Med.*, **298**, 1321.

SLOCUM, H. K., PAVELIC, Z. P. & RUSTUM, Y. M. (1980) An enzymatic method for the disaggregation of human solid tumors for studies of clonogenicity and biochemical determinants of drug action. In *Cloning of Human Tumor Stem Cells* (Ed. Salmon). New York: Alan R. Liss, Inc. p. 339.

SOEHNLEN, B., YOUNG, L. & LIN (1980) Standard laboratory procedures for *in vitro* assay of human tumour stem cells. In *Cloning of Human Tumour Stem Cells* (Ed. Salmon) New York: Alan R. Liss, Inc. p. 331.